# Intravascular NK/T-cell lymphoma, Epstein–Barr virus positive with multiorgan involvement: a clinical dilemma

**DOI:** 10.1186/s12885-018-5001-6

**Published:** 2018-11-15

**Authors:** Magda Zanelli, Maria Cecilia Mengoli, Rachele Del Sordo, Angelo Cagini, Loredana De Marco, Edoardo Simonetti, Giovanni Martino, Maurizio Zizzo, Stefano Ascani

**Affiliations:** 1 Pathology Unit, Azienda Unità Sanitaria Locale/IRCCS di Reggio Emilia, Reggio Emilia, Italy; 2 Department of Experimental Medicine, Medical School, Section of Pathological Anatomy and Histology, University of Perugia, Perugia, Italy; 3 Hematology Unit, Università degli Studi di Perugia, CREO Perugia, Perugia, Italy; 4 Surgical Oncology Unit, Azienda Unità Sanitaria Locale/IRCCS di Reggio Emilia, Reggio Emilia, Italy; 5 Clinical and Experimental Medicine PhD Program, University of Modena and Reggio Emilia, Modena, Italy; 6 Pathology Unit, Ospedale di Terni, University of Perugia, Perugia, Italy

**Keywords:** Intravascular, Lymphoma, Natural killer cell, T-cell, Epstein-Barr virus

## Abstract

**Background:**

Intravascular lymphoma is a rare type of non-Hodgkin lymphoma mostly of B-cell lineage. A few cases of intravascular lymphoma have been found to be of NK/T-cell origin, mainly affecting the skin and central nervous system.

**Case presentation:**

A 54-year-old Caucasian man sought care because of a 2 weeks history of jaundice and intermittent fever, not responsive to antibiotics and antipyretics. Laboratory tests showed low blood oxygen concentration and pancytopenia. Serum microbiological tests were negative. Computerized tomography (CT) scan revealed hepatosplenomegaly and diffuse ground-glass opacities in both lungs without interlobular septal thickening. Despite oxygen therapy, the clinical conditions rapidly deteriorated leading to death 3 days after admission. Autopsy revealed a multiorgan involvement by an Epstein-Barr virus positive NK/T-cell lymphoma, strikingly growing within the blood vessel lumina, in absence of skin lesions.

**Conclusions:**

The current case highlights the pathological features of this rare entity, the protean clinical presentation of which is often misleading, resulting in delayed diagnosis and treatment.

## Background

Intravascular lymphoma (IVL) represents a rare neoplasm in which the tumor cells are confined to the lumina of blood vessels. Skin and central nervous system (CNS) are most commonly involved, although virtually any organ can be affected [1]. The majority of cases are of B-cell origin [1], however, rare cases of T and natural killer (NK) immunophenotype have been reported [2–18]. IVL of B-cell phenotype are rarely Epstein-Barr virus (EBV) positive [8], whereas the frequent detection of EBV in NK/T cell IVL supports its possible pathogenic role. NK/T-cell IVL differs from nasal-type NK/T-cell lymphoma mainly by its intravascular nature. NK/T-cell IVL is an aggressive neoplasm with a poor outcome, although the prognosis appears to be related to the extent of disease.

Herein we report the case of a 54-year-old male with a rapidly progressive course. The clinical picture was somewhat confusing and led the physicians to suspect a generalized sepsis. Autopsy disclosed involvement of multiple organs by IVL NK/T-cell lymphoma, in absence of skin lesions. A review of the reported cases of NK/T-cell IVL with immunohistochemical and molecular data is also performed.

## Case presentation

A 54-year-old Caucasian man sought care because of a 2 weeks history of jaundice and intermittent fever (up to 39 °C), not responsive to antibiotics and antipyretics. His past medical records included arterial hyperthension and a left vertebral artery dissection. Upon admission, he was pyretic, jaundiced, tachypneic and lypotimic. No cutaneous lesions were present. Neurological examination was normal. Laboratory tests showed low blood oxygen concentration (pO_2_ 62 mmHg, pCO_2_ 22 mmHg, HCO_3_ 18.8 mmol/L, pH 7.55), anemia (Hb 10.2 g/dL), leukocytopenia (3.100/mcL) and thrombocytopenia (62.000/mcL). Atypical circulating lymphocytes were absent. Increased levels of transaminases (ALT 1374 u/L; AST 654 u/L), gamma-GT (802 u/L) and lactate dehydrogenase (LDH 2998 u/L) were present. Serum microbiological tests were negative. Computerized tomography (CT) scan revealed hepato-splenomegaly and diffuse ground-glass opacities in both lungs without interlobular septal thickening. No lesion was detected in the upper aerodigestive tract. Despite oxygen therapy, the clinical conditions rapidly deteriorated leading to death 3 days after admission. A severe, generalized sepsis was suspected. A total-body autopsy was performed.

**Fig. 1:**
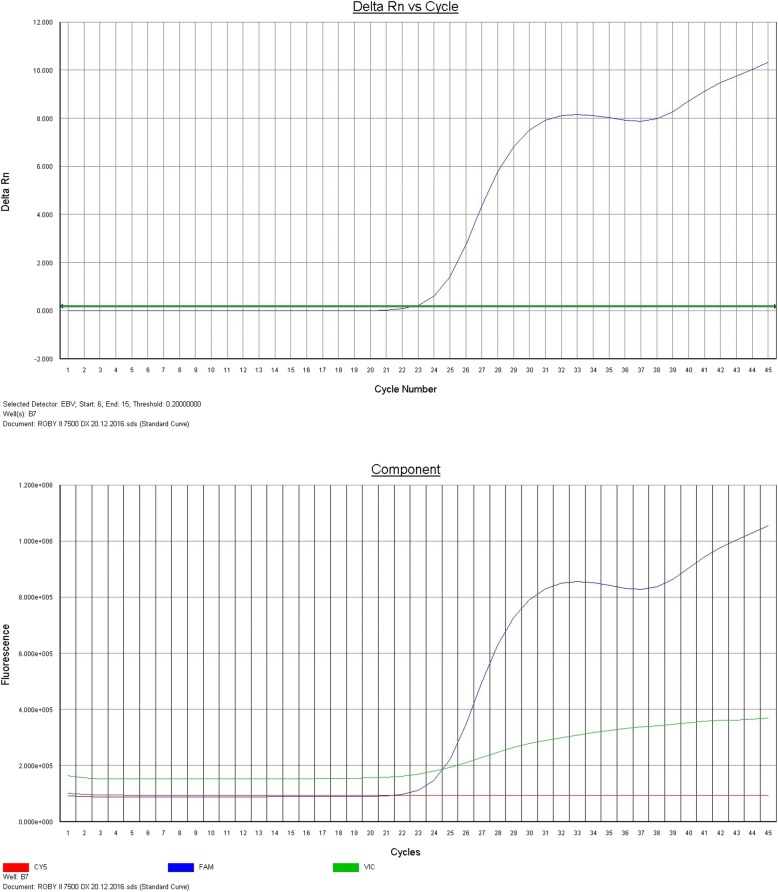
EBV DNA detection in pleural effusion by Polymerase chain reaction (PCR)

Gross examination revealed pericardial, pleural and peritoneal effusions. The lungs were heavier than normal (right lung 910 g; left lung 930 g) with multiple foci of consolidation. The spleen was enlarged (610 g) as well as the liver (1920 g), without focal lesions. No lesions were found in the skin, oral cavity or oropharynx. Polymerase chain reaction (PCR) detected about 2 millions copies of EBV DNA on pleural (Fig. 1) effusion and lung tissue.

**Fig. 2:**
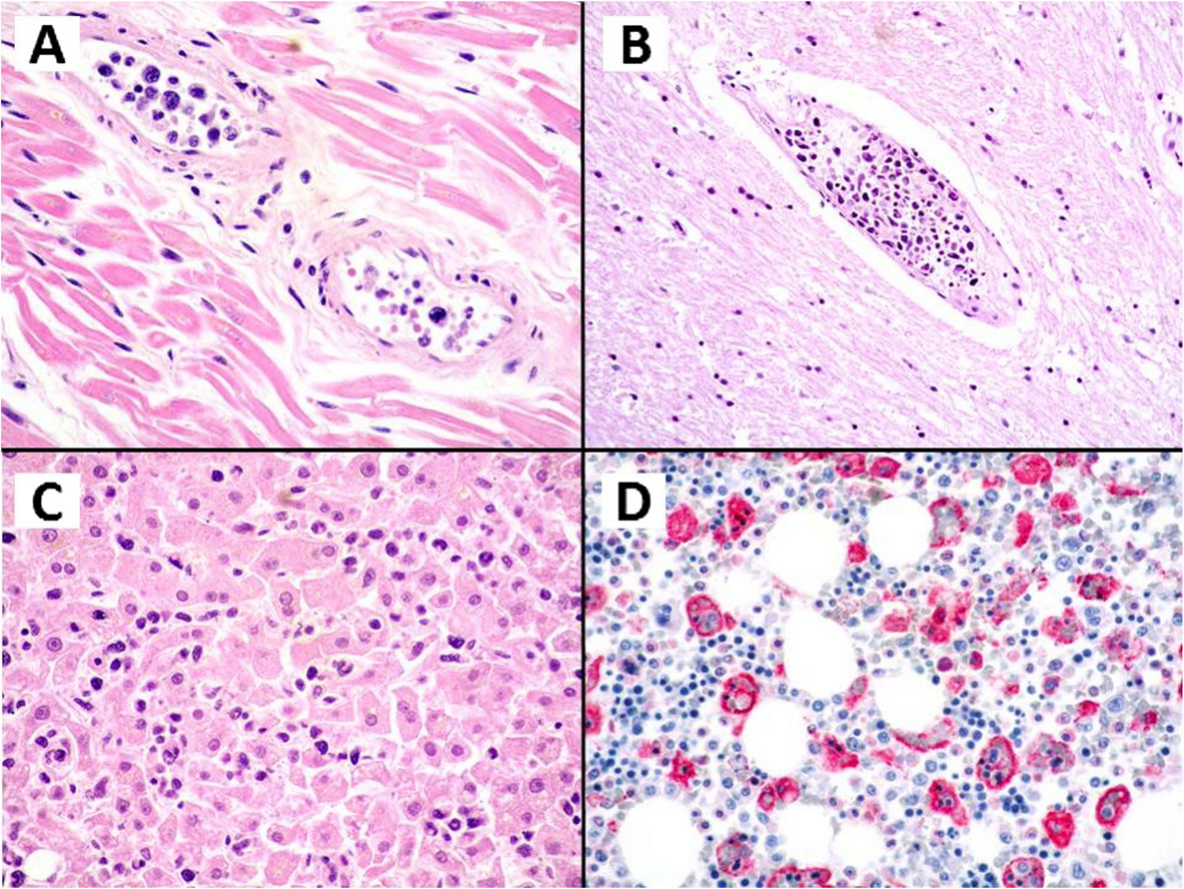
Haematoxylin and Eosin, medium power of view of the myocardium (**a**), of the brain (**b**) and liver parenchyma (**c**), show a prominent intravascular growth of neoplastic, pleomorphic cells without invasion of the organs parenchyma characteristic of intravascular lymphoma either of B or NK/T lineage. Immunohistochemistry with CD68 PGM1 (**d**) of the bone marrow evidence prominent features of haemophagocytotosis

**Fig. 3:**
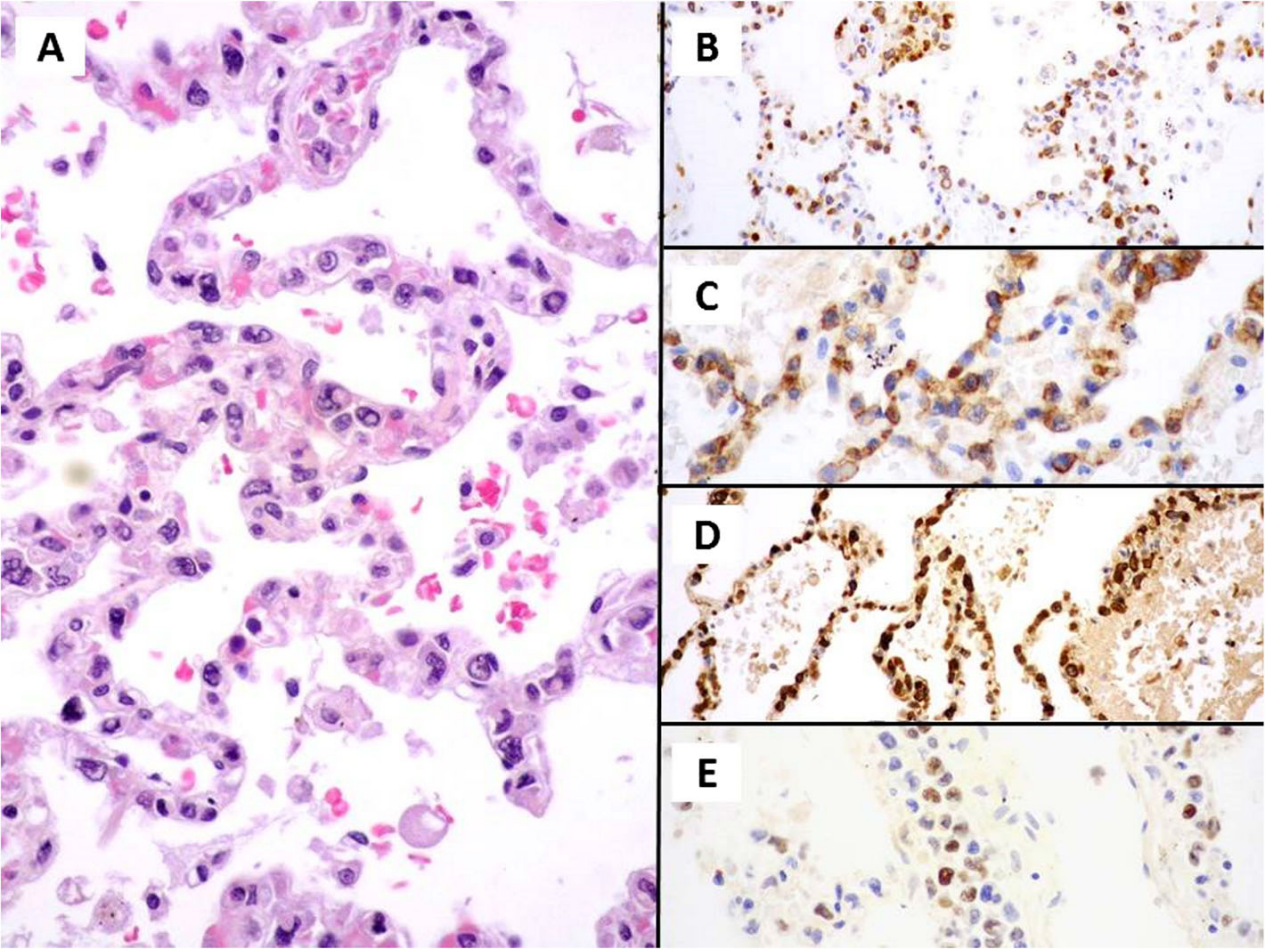
Haematoxylin and Eosin, medium power of view of the lung parenchyma with striking features of growth of the neoplastic cells within the capillaries of the alveolar walls (**a**). Immunohistochemistry with CD3 (**b**), CD56 (**c**) and granzyme B (**d**) demonstrate a positive staining of the neoplastic intravascular cells. EBER (**e**) has been demonstrated also to be diffusely positive in this case of intravascular NK/T lymphoma

**Fig. 4:**
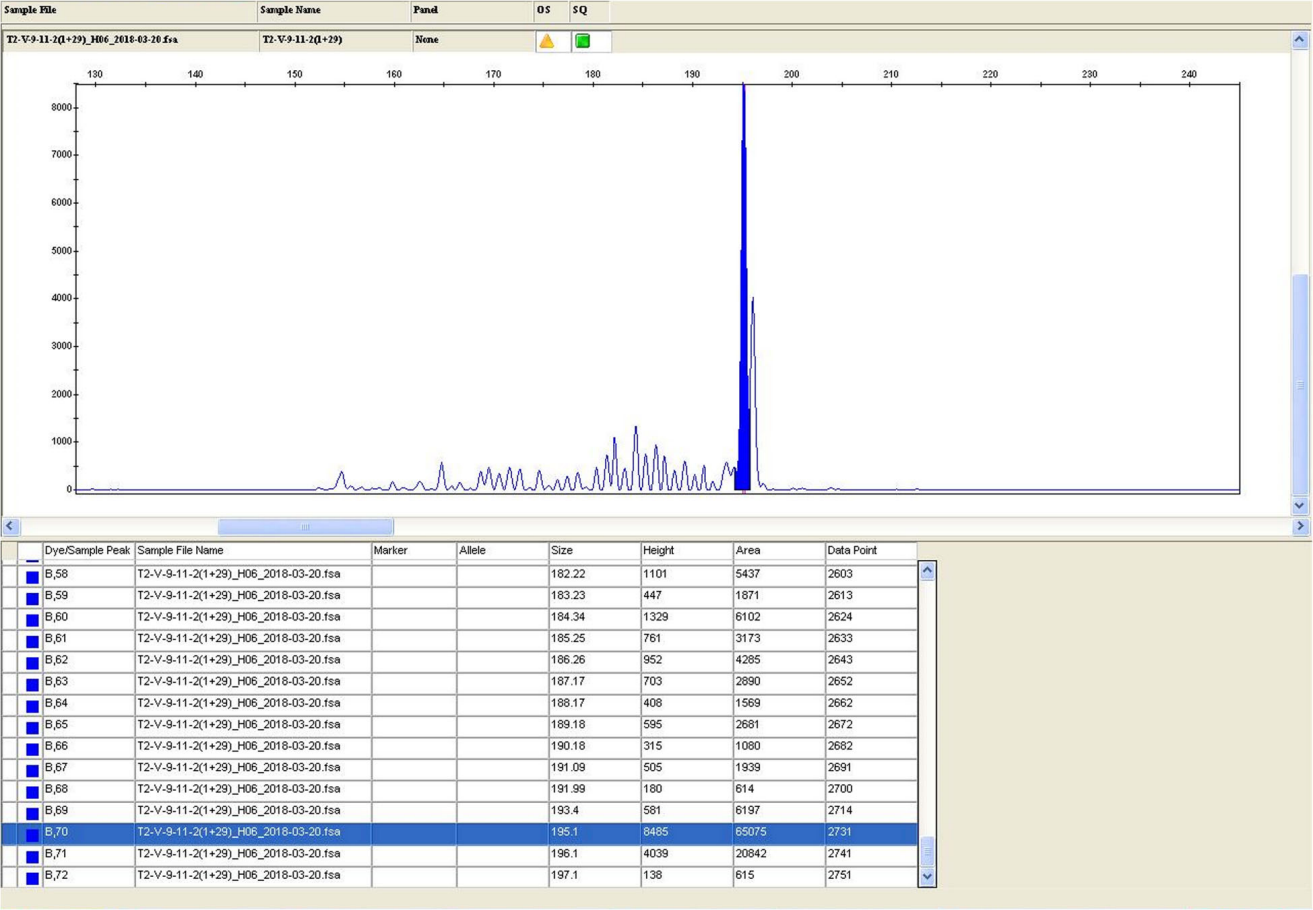
TCR assay provided molecular genetic evidence of clonality. One reproducible clonal peak is showed in TCR-G

Histology revealed atypical lymphoid cells filling and expanding the lumina of small and medium blood vessels (Fig. 2) in virtually any organs (heart, lung, kidney, spleen, liver, brain). A sinusoidal involvement occurred in bone marrow, with about 10% of neoplastic infiltrate. Features of hemophagocytosis (Fig. 2d) were also evident in bone marrow. The lymphoid cells, strikingly confined to the blood vessels lumina, were large-sized with hyperchromatic nuclei and expressed at immunohistochemistry: CD3 (Fig. 3b), CD2, perforin, CD56 (Fig. 3c), granzyme B (Fig. 3d), showing a T and cytotoxic phenotype. CD20, CD79alfa, PAX5, CD4, CD8, CD5, ALK1, CD16 were all negative. Either external or internal positive controls were used to validate the assay for each immunohistochemical staining. The proliferative index (Ki67) was high (approximately of 80%). EBV encoded RNA in situ hybridization (EBER) was diffusely positive (Fig. 3e). Polymerase chain reaction identified a clonal T-cell receptor gamma gene rearrangement (Fig. 4). The final diagnosis was EBV positive intravascular NK/T-cell lymphoma with multisystem involvement.

## Discussion and conclusions

IVL are neoplasms, mostly of B-cell phenotype, characterized by the presence of large lymphoid cells strikingly restricted to the blood vessels lumina.

Intravascular large B-cell lymphoma is recognized as a distinct lymphoproliferative disorder by World Health Organization (WHO) [1]. Two patterns of presentation have been recognized: a classic, Western variant involving more often skin and CNS and an Asian variant presenting with multiorgan failure, often associated with hemophagocytic syndrome [19]. Cases limited to the skin usually have a better prognosis.

IVL of T and NK-cell lineage are exceedingly rare and very few cases have been reported so far [2–18]. NK/T-cell IVL is still not recognized as a specific entity by the last WHO classification of lymphoid neoplasms, probably due to its rarity. Extranodal NK/T-cell lymphoma, nasal-type rarely manifests features of intravascular lymphoma, involving sites such as the skin and CNS [1]. The main differential diagnosis of NK/T IVL includes: extranodal NK/T-cell lymphoma, nasal type and aggressive NK-cell leukaemia, both of which are EBV-related, and primary cutaneous anaplastic CD30 positive T-cell lymphoma. Neoplastic cells are confined within lymphatic vessels in cutaneous anaplastic CD30 positive T-cell lymphoma, its course is indolent and EBER is negative [1].

In nasal-type NK/T-cell lymphoma, the upper aerodigestive tract is the privileged site of involvement, although other extranodal sites, including the skin, have been reported [1]. The neoplastic cells permeate the tissues with angioinvasion and are not limited to the endovascular system. Aggressive NK-cell leukaemia is considered the leukaemic counterpart of nasal-type NK/Tcell lymphoma and a leukemic blood picture is usually present, distinguishing it from intravascular NK/T-cell lymphoma [1]. CD16 positivity is frequently seen in aggressive NK-cell leukemia, whereas both extranodal NK/T-cell lymphoma, nasal-type and intravascular NK/T-cell lymphoma show CD16 negativity, as it was in our case.

To the best of our knowledge, only 24 cases of NK/T-cell IVL with sufficient immunohistochemical and molecular data, including the present case, have been reported so far [2–18]. The clinicopathologic features are summarized in Table 1. The patients, 11 males and 13 females, ranged from 18 to 87 years of age. The skin and CNS were frequently involved, although any organ can be affected. The disease often presented with skin lesions (22/24) and in 11 cases the skin was the unique site of involvement. Sharma et al. reported a case presenting with almost unique brain involvement [17]. In 5 patients the disease affected both skin and brain [2, 5, 7, 12, 16]. Neurologic symptoms (depression and dizziness), reflecting possibly brain involvement, occurred in 2 more cases, concurrently with skin lesions [7, 9]. Two patients had skin lesions and manifestations in other organs (ileum plus spleen [6] and liver [14], respectively). In one report, in addition to skin lesions, the patient had portal hypertension of unknown origin, thought to be possibly related to liver involvement [7]. However, in absence of post-mortem examination, it is always presumable that internal organs might have had undetected involvement.

**Table 1:** Demographic data, clinical data, and characteristics of reported cases of IVL of T and NK-cell lineage

Ref.	Age/sex	Sites involved at presentation/(symptoms/ signs)	Sites involved during disease course	BOM involvement	Immunophenotype	EBER	TCR	Therapy/outcome
Santucci 2003 [2]	54/M	Skin lesions, weight loss, leukopenia	CNS	NA	CD3e + CD56 + TIA-1 + GrB + CD30 + ki671 + 100%LMP1-CD4- CD8- CD20- CD79a- CD57- CD68- Bcl2-	+	NA	CHOP/Exitus 17 mo after diagnosis
Wu 2005 [3]	41/M	Skin lesions	none	–	CD3e + CD2 + CD7 + CD56 + CD43 + perforin+ TIA-1 + Bcl2 + CD20- CD4- CD8- CD5- TCRbF1- CD30- lysozyme- MPO- Keratin-	+	TCR germline	CHOP+ SCT CR at 12 mo
Wu 2005 [3]	47/F	Fever, weakness, arthralgia, myalgia, mental signs+ pancytopenia	Disseminated disease at autopsy (brain, bone marrow, kidneys, ovaries, cervix)	+ (diagnosis made on BOM biopsy)	CD3e + CD2 + CD7 + CD56 + GrB + TIA-1 + CD20- CD5- CD4- CD8- CD57-	–	TCR germline	Unspecified therapy/exitus 15 days after diagnosis
Kuo 2006 [4]	71/F	Skin lesions	none	–	CD3e + CD56 + TIA1 + KI67 + 90%CD4- CD5- CD8- CD10- CD20-CD30- BCL6- LMP-1-	+	TCR germline	No therapy/alive at 5 mo
Song 2007	40/F	Skin lesions+CNS	none	–	LCA + CD3 + CD56+ GrB + TIA + 1+ Ki67 + 100% CD20- CD4- CD8- CD29-	+	TCR germline	CODOX-M + IVAC/alive at 7mo
Nakamichi 2008 [6]	23/F	Skin lesions	Ileum, spleen+fever	–	CD3e + CD56+ TIA1 + ki67 + 100%CD20- CD79a- CD45RO-	+	TCR germline	CHOP followed by PromMACE/CYTABOM, L-ASP/CY, hyper CVAD/MTX-AraC, SCT. Exitus after 9 mo, due to aGVHD
Cerroni 2008 [7]	67/F	Skin lesion+CNS	none	NA	CD2+ CD3e + CD8 + CD56-TIA-1 + CD4- CD20- CD30+/- CD56-	NA	TCR monoclonal (TCRg)	Exitus after 7 days
Cerroni 2008 [7]	63/M	Skin lesions+arthralgias, weight loss, fever, depression+leukopenia+ thrombocytopenia	none	NA	CD2 + CD3e + TIA1 + CD56+ CD4- CD5- CD7- CD8- CD20- CD45RO + bF1-	+	TCR germline	Exitus after 6 mo
Cerroni 2008 [7]	64/M	(History of B-CLL). Skin lesions	NA	- (B-CLL+)	CD2 + CD3e + TIA-1 + CD4- CD5- CD8- CD20- CD56- bF1-	–	TCR germline	CHOP, exitus after 7 mo
Cerroni 2008 [7]	87/M	Skin lesions+portal hyperthension	none	NA	CD2 + CD3e + CD56- TIA-1- GrB-perforin- CD4- CD5- CD7- CD8-CD20- CD30- CD45RO- bF1-	+	TCR monoclonal (TCRg)	Exitus after 15 days
Gleason 2008 [8]	62/M	Skin lesions+night sweats	none	–	LCA + CD2 + CD3e + CD43 + CD56 + Perforin+ GrB + TIA-1 + CD4- CD5- CD7- CD8- CD20- CD79a- BCL2- CD30- CD45RO- TCRbF1- TdT- MPO- CK- CD34-	–	TCR monoclonal(TCRg)	CHOP followed by DHAP. AWD at 8 mo
Liao 2011 [11]	42/F	Skin lesions+ malaise, dizziness.	none	–	CD3e + CD56 + Gr-B + KI67 + 99%,bF1- CD4- CD5- CD8- CD20- CD30- PAX5- TdT-	+	NA	RT + CT: (CHOP,Bortezomib, EPOCH); alive at 14 mo
Yanning 2013	84/F	Skin lesions+fever, weight loss	NA	NA	CD2 + CD3e + CD45RO + GrB + CD4- CD5- CD7- CD8- CD20- CD79a- CD56- CD30- panCK-	+	TCR germline	Therapy refused. AWD at 4 mo
Jang 2014	23/F	Skin lesions	none	–	CD3 + CD8 + GrB + TIA-1 + LCA + MPO + CD4- CD5- CD20- CD30- CD56- CK-	+	TCR germline IgH gene clonal	CHOP+intratecal MTX + PBSCT. Skin recurrence followed by unspecified CT. Exitus 11 mo after recurrence
Liu 2014	38/F	Skin lesions	CNS	–	CD3 + CD56 + GrB + Ki67 + 90%CD4- CD5- CD8- CD20- CD30- PAX5- TdT-	+	NA	CHOP. After 7 mo recurrence at CNS. Exitus 13 mo after diagnosis
Wang 2015	45/M	Skin lesions+malaise, fever, weight loss	NA	NA	CD2 + CD3e + perforin+ GrB + TIA1 + CD56 + CD4- CD8- CD20- TCRb- TCRg- CD30- KI67 + 90–100%	+	TCR germline	Exitus 15 days after diagnosis
Wang 2015	32/F	Skin lesions+fever	none	NA	CD2 + CD3e + perforin+ GrB + TIA1 + CD56 + CD4- CD8- CD20- TCRb- TCRg- CD30- KI67 + 90–100%	+	TCR germline	CHOP. Exitus 4 mo after diagnosis
Wang 2015	18/F	Skin lesions	none	–	CD2 + CD3e + perforin+ GrB + TIA1 + CD56 + CD4- CD8- CD20- TCRb- TCRg- CD30 + KI67 + 90–100%	+	TCR germline	CHOP. Alive 3 yrs. after diagnosis
Bi 2015	29/M	Skin lesions, fever, weight loss	Liver	–	CD3 + CD43 + CD56 + TIA-1 + CD30 + CD4- CD5- CD7- CD8- CD20- CD79a- KI67 + 90%	+	TCR germline	HyperCVAD. Exitus 3 mo after diagnosis
Alhumidi 2015	48/F	Skin lesions	None	–	CD45 + CD3 + GrB + CD56- CD4- CD5- CD8- CD20-	+	NA	CT. Alive 18 mo after diagnosis
Jaffe 2017	51/M	Skin lesions+CNS	none	NA	CD3 + CD8 + TIA-1 + GrB + CD20- CD79a- CD56- ALK-1-	+	TCR monoclonal (TCR-g)	NA
Sharma 2017	62/F	CNS	none	+ on molecular analysis (TCR)	CD3 + CD8 + CD57 + GrB + TIA-1 + CD2- CD4- CD5- CD7- CD30- CD56- TdT- PAX5-	–	TCR monoclonal	Methylprednisolone, dexamethasone, intratechal MTX, cytarabine. Exitus
Alegria-Landa 2017	81/M	Skin lesions	none	–	CD3 + GrB + perforin+ CD30 + CD20- CD4- CD8- TCRb- TCRg- CD56-	+	TCR monoclonal (TCRbTCRg)	Exitus 15 days after diagnosis
Present case	54/M	Jaundice, fever, respiratory symptoms, pancytopenia. PB: no atypical lymphocytes	Disseminated disease at autopsy (brain, heart, kidney, lung, bone marrow)	+ (subtle infiltration)	CD3 + CD2 + perforin+ GrB + CD56 + KI67 + 80% CD20- CD79a- PAX5- CD4- CD8- CD5- ALK1-CD16-	+	TCR monoclonal	Exitus 18 days after presentation

*F*female, *M* male, *BOM* bone marrow, *NA* not assessed, *CR* complete remission, *mo* months, *yrs*. years, *B-CLL* B chronic lymphocytic leukemia, *RT* radiotherapy, *CT* chemotherapy, *CHOP* cyclophosphamide, doxorubicin, vincristine, prednisone, *SCT* Stem cell transplant, *CODOX-M* cyclophosphamide, vincristine, doxorubicin, methotrexate, *IVAC* ifosfamide, mesna, etoposide, cytarabine, *ProMACE/CytaBOM* prednisone, methotrexate, doxorubicin, cyclophosphamide, etoposide, cytosine, arabinoside, bleomycin, vincristine, leucovorin, *l-ASP* l-asparaginase, *CY* cyclophosphamide, *hyper-CVAD* hyperfractionated cyclophosphamide, vincristine, doxorubicin, dexamethasone, *MTX* methotrexate, *AraC* cytosine arabinoside, *aGVHD* acute graft-versus-host disease, *EPOCH* etoposide, prednisolone, vincristine, cyclophosphamide, doxorubicin, *DHAP* dexamethasone, cytarabine, cisplatin, *PBSCT* peripheral blood stem-cell transplant, *PB* peripheral blood, *AWD* alive with disease

Wang et al. [13] reported 5 cases of NK/T-cell IVL, but, as commented by Alegria-Landa et al. [8] in 2017, the possibility of an extranodal NK/T-cell lymphoma nasal-type in their case number 2 cannot be excluded. As well in their case number 5, the presence of circulating atypical CD56 positive lymphocytes, makes aggressive NK leukemia the most likely diagnosis. In the present review these 2 cases have been discounted.

Protean clinical findings, including fever, malaise, weight loss, arthralgia, night sweats and jaundice were reported half of time (11 cases). The confusing clinical picture might be misleading, as demonstrated in the current case, compromising a prompt diagnosis and treatment. Hematologic findings, including leukopenia, thrombocytopenia, pancytopenia occurred in 4 patients as well as in our case [2, 3, 7]. Bone marrow involvement is uncommon, being detected in 3/16 evaluated, including the present report [3, 17]. In the case by Wu et al. the diagnosis was made on bone marrow biopsy in a 47-year-old female with protean systemic and neurological symptoms [3]. As Wu et al. pointed out the lymphomatous sinusoidal infiltrate in bone marrow might be subtle (as in our case) and detected only by immunohistochemical staining [3]. In the case of Sharma et al. bone marrow involvement was identified only by molecular studies showing a clonal T-cell population [17].

Clinically our case shows some similarities with case number 2 reported by Wu [3]. In both cases skin lesions were strikingly absent (differently from all the other cases reported) and the disease was widely disseminated at presentation, as confirmed at post mortem examination. A subtle bone marrow involvement was identified in both cases. In addition, in the present report features of hemophagocytosis were also evident. In both cases the disease’s course was rapid and fatal.

Most NK/T-cell IVL share a common profile with a cytotoxic phenotype (CD56 and cytotoxic molecules positivity), although the expression of individual immunohistochemical markers may change from case to case.

A strong association with EBV was evident as EBER positivity was detected in 19 out of 23 tested diseases. The frequent association with EBV infection underlines the similarities with NK/T-cell lymphoma nasal-type. Thirteen of 20 evaluated cases had a germline configuration of TCR gene. In the remaining 7 cases, including the present report, a monoclonal TCR gene rearrangement was identified.

As already mentioned, NK/T-cell IVL can be difficult to diagnose due to its multifaceted clinical presentation. It can involve any organ, although more frequently CNS and skin. In addition to non-specific symptoms (fever, weight loss, malaise), patients commonly present skin lesions (as erythematous, indurated plaques and nodules) and often bizarre neurologic symptoms, caused by multiple sites of infarct resulting from vascular occlusion. The pathological diagnosis of NK/T-cell IVL can be established by combined histopathologic, immunohistochemical and molecular studies. However, it has to be stressed that, in case of a small number of neoplastic cells in vessels, NK/T-cell IVL can be easily overlooked. Therefore, a strict clinicopathological correlation is essential to get to a prompt diagnosis.

NK/T-cell IVL often follows an aggressive clinical course, however, the prognosis may vary with the extent of disease, being more favorable in patients with exclusive skin lesions. In particular 2 cases with the disease limited to the skin had a really favorable outcome, being in complete remission after 1.5 and 3 years [13, 15]. However, the limited number of cases makes difficult to draw any conclusion. The literature data suggest that CHOP regimen is insufficient for the treatment of NK/T-cell IVL, whereas a combination of intensive chemotherapy often anthracycline-based and stem cell transplantation may improve the outcome [6, 12].

In summary, presenting this case of NK/T-cell IVL with multiorgan involvement and lacking the more common skin presentation, we would like to draw attention on a very rare entity of which both clinicians and pathologists should be aware. As the clinical picture can be very misleading, a high level of suspicion is essential to achieve a prompt diagnosis.

## Abbreviations

EBV: Epstein-Barr virus
IVL: Intravascular lymphoma
NK: Natural killer
PCR: Polymerase chain reaction
WHO: World Health Organization
